# Cerebral small vessel disease: Pathological mechanisms and potential therapeutic targets

**DOI:** 10.3389/fnagi.2022.961661

**Published:** 2022-08-12

**Authors:** Yue Gao, Di Li, Jianwen Lin, Aline M. Thomas, Jianyu Miao, Dong Chen, Shen Li, Chengyan Chu

**Affiliations:** ^1^Department of Neurointervention and Neurological Intensive Care, Dalian Municipal Central Hospital, Dalian, China; ^2^Department of Neurology, Dalian Municipal Central Hospital, Dalian, China; ^3^F. M. Kirby Research Center for Functional Brain Imaging, Kennedy Krieger Institution, Baltimore, MD, United States; ^4^Department of Neurosurgery, Dalian Municipal Central Hospital, Dalian, China; ^5^Department of Neurology and Psychiatry, Beijing Shijitan Hospital, Capital Medical University, Beijing, China

**Keywords:** cerebral small vessel disease, cognitive impairment, endothelial dysfunction, blood-brain barrier breakdown, white matter change, inflammation

## Abstract

Cerebral small vessel disease (CSVD) represents a diverse cluster of cerebrovascular diseases primarily affecting small arteries, capillaries, arterioles and venules. The diagnosis of CSVD relies on the identification of small subcortical infarcts, lacunes, white matter hyperintensities, perivascular spaces, and microbleeds using neuroimaging. CSVD is observed in 25% of strokes worldwide and is the most common pathology of cognitive decline and dementia in the elderly. Still, due to the poor understanding of pathophysiology in CSVD, there is not an effective preventative or therapeutic approach for CSVD. The most widely accepted approach to CSVD treatment is to mitigate vascular risk factors and adopt a healthier lifestyle. Thus, a deeper understanding of pathogenesis may foster more specific therapies. Here, we review the underlying mechanisms of pathological characteristics in CSVD development, with a focus on endothelial dysfunction, blood-brain barrier impairment and white matter change. We also describe inflammation in CSVD, whose role in contributing to CSVD pathology is gaining interest. Finally, we update the current treatments and preventative measures of CSVD, as well as discuss potential targets and novel strategies for CSVD treatment.

## Introduction

Cerebral small vessel disease (CSVD) refers to a disorder of perforating cerebral arterioles, capillaries, and venules (Wardlaw et al., 2013b). Typical magnetic resonance imaging (MRI) markers of CSVD in the brain include white matter hyperintensities (WMHs), lacunes, microbleeds, enlarged perivascular spaces, and subcortical infarcts (Wardlaw et al., 2013c). CSVD was once thought to be innocuous with clinically silent manifestations, but is now recognized as a major risk factor for stroke, present in about a quarter of ischemic strokes and most hemorrhagic strokes (Kapasi et al., 2017; Wardlaw et al., 2019). CSVD is also associated with gait problems and mood disturbances in older people (Choi et al., 2012; Wardlaw et al., 2013c). Furthermore, CSVD has become the leading vascular contributor to cognitive impairment and dementia worldwide, posing a massive burden to societies and health-care systems world-wide facing increased life expectancies and a more aged population (Gorelick et al., 2011).

Although the importance of and concern regarding CSVD is clearly recognized, there is a lack of effective prophylactic or therapeutic regimens. The current approach is management of vascular risk factors associated with CSVD including hypertension, smoking, diabetes, and hypercholesterolemia (Wardlaw et al., 2014, 2019). In fact, the failure in achieving a breakthrough for CSVD treatment is primarily due to a poor understanding of its etiology. Initially, a compromised blood-brain barrier (BBB) integrity was postulated to create both microbleeds and reduced blood flow distally that would induce ischemia and the subcortical infarcts (Wardlaw et al., 2003). These ischemic changes may in turn lead to the loss of oligodendrocytes, contributing to the impaired myelination in CSVD (Peters, 2007), which corresponds to the WMHs on MR images. However, a subsequent study suggested that endothelial dysfunction is the key initiator for CSVD and its pathogenesis, predating BBB breakdown (Rajani et al., 2018). Recently, the contribution of inflammation to CSVD has attracted increasing attention and an emerging concept of “inflammaging” referring to the chronic, sterile, low-grade inflammation observed in older organisms and humans, and brain inflammaging was proposed as an etiological factor for CSVD (Li T. et al., 2020).

Given the multifactorial and complex nature of CSVD, a better understanding of its pathogenesis would aid in the successful development of specific and effective interventions for CSVD. Hence, in this review we will integrate the pathophysiological events of the endothelium, smooth muscle cells, BBB, neural cells and inflammation and provide their possible linkages for more insight into CSVD pathogenesis. In addition, we will discuss the current treatments as well as potential targets to prevent and repair or even reverse the brain damage in CSVD.

## Mechanistic insights into pathological alterations in cerebral small vessel disease

### Endothelial dysfunction

**FIGURE 1:**
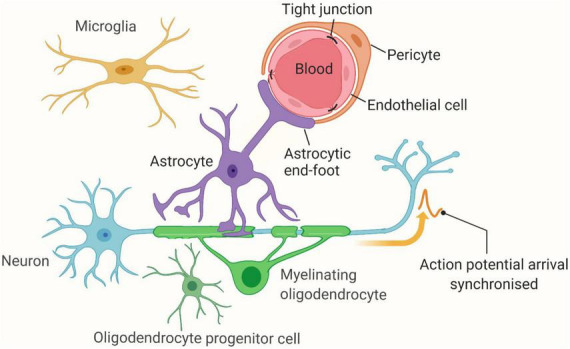
The Neurovascular Unit (NVU). Vascular (endothelial cells and pericytes) and brain (astrocytes, oligodendrocytes, and neurons) cells interact and function as a unit, millions of which reside in the brain. Figure created using BioRender.com.

Endothelial cells (ECs) serve as a functional and structural barrier between tissue and blood, modulating blood flow, regulating transport of circulating components and participating in inflammatory processes. In the brain tissue, ECs have an additional role as a vital part of the BBB and the neurovascular unit (NVU) (Figure 1). Therefore, the alteration of ECs in function or quantity was initially believed to be an etiological contributor to CSVD. Several studies have revealed that brain ECs remain microscopically intact and that cellular loss is not observed even in cases with severe CSVD (Lammie, 2002; Craggs et al., 2013; Hainsworth et al., 2015). More recently, increasing evidence has emerged suggesting that endothelial dysfunction may initiate pathological changes, both in genetic and sporadic CSVD (Quick et al., 2021).

**FIGURE 2:**
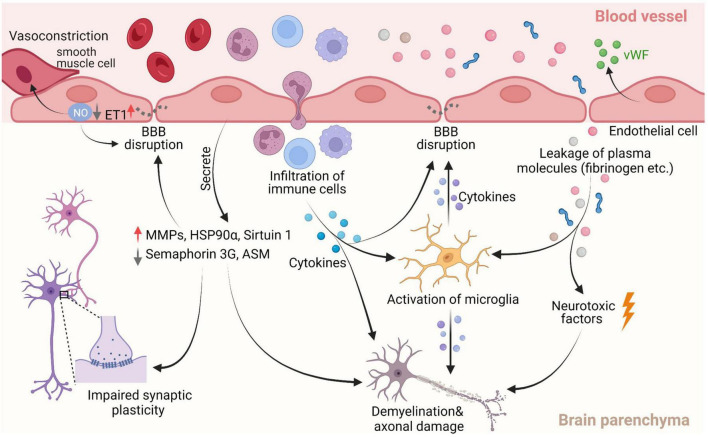
Pathological characteristics of cerebral small vessel disease (CSVD). Of them, decreased nitric oxide (NO) bioavailability is the most commonly used marker of dysfunction in endothelial cells (ECs). Reducing NO levels can lead to less vasodilation of neighboring smooth muscle cells, which in turn causes nitrosylation or nitrosation of tight junction (TJ) proteins, ultimately resulting in BBB disruption. Increased ET1 release by dysfunctional ECs can give rise to pathological vasoconstriction. The reduction of semaphorin 3G and sirt1 due to endothelial dysfunction can affect synaptic plasticity and BBB integrity respectively, and consequently, contribute to cognitive decline. Additionally, the factors secreted by dysfunctional ECs such as von Willebrand factor (vWF), heat shock protein 90α (HSP 90α), acid sphingomyelinase (ASM) and matrix metalloproteinases (MMPs) can induce vessel damage directly, as well as impair the integrity of the BBB and white matter. Compromising the BBB allows for entry of potential detrimental plasma components and immune cells into the brain parenchyma, further promoting BBB breakdown and white mater damage *via* neuroinflammation and direct neurotoxic effects. Figure created using BioRender.com.

Endothelial dysfunction-induced brain damage manifests in several ways *via* different mechanisms (Figure 2). In CSVD patients, both impairment and autoregulation of cerebral blood flow (CBF) were observed using positron emission tomography (PET) and MRI, respectively (Poggesi et al., 2016). ECs are undeniably involved in this pathological change as one of the primary roles of ECs is to regulate the vascular tone of the vessel wall in cerebral arteries and microvessels. EC-derived nitric oxide (NO) is an important signaling molecule for local CBF regulation that mediates vessel dilation in response to external stimuli (Katusic and Austin, 2014; Hu et al., 2017; De Silva and Faraci, 2020). Reduced release of NO is an established metric for detecting endothelial dysfunction, as its occurrence has been reported to result in pathological vasoconstriction, compromised CBF, and ultimately, tissue ischemia (Gunarathne et al., 2009; Quick et al., 2021). The bioavailability of NO can be experimentally measured using the Griess reaction; however, a clinically relevant version of this method is not yet available. In patients, the relative change in reflective index (ΔRI%) upon administration of an NO-releasing β2-andrenergic receptor stimulant agent such as Salbutamol can be used to evaluate endothelial function (Gunarathne et al., 2009). The level of endothelial NO synthase (eNOS), an enzyme that converts L-arginine to NO, can be used to evaluate the production of NO as well. Studies in CSVD animal models and of the cerebrospinal fluid (CSF) of CSVD patients have reported reduced eNOS, indicating EC dysfunction (Quick et al., 2021). Additionally, vascular risk factors such as aging and hypertension, can raise the level of reactive oxygen species, driving eNOS to produce the destructive superoxide anion (O_2_^–^), which in turn further degrades bioavailability of NO (Villamor et al., 2003; De Silva and Faraci, 2020). Endothelin-1 (ET1) is an important regulating factor for maintaining homeostasis in the vessel, which can act on smooth muscle cells to cause vasoconstriction, but also can stimulate adjacent ECs to produce NO to cause vasodilation (Houde et al., 2016). EC dysfunction results in elevated ET1 in plasma and disrupts the balance between NO and ET1, making ET1 another contributor to the pathological vasoconstriction (Galatius et al., 1999; Quick et al., 2021).

Semaphorin 3G, a recently characterized member of the class 3 secreted semaphorin subfamily, is heavily expressed in ECs, but not in neuronal or glial cells. It has been shown that Semaphorin 3G is an endothelium-derived synaptic organizer, whose loss in ECs resulted in behavioral and memory deficits (Zhang et al., 2014; Tan et al., 2019). This study demonstrated a critical role for semaphorin 3G in regulating synaptic function in the hippocampus *via* the Nrp2/PlexinA4 signaling cascade as well (Tan et al., 2019). Thus, it is speculated that ECs dysfunction may promote cognitive decline in CSVD *via* non-vascular effects on synaptic organization, in addition to vascular effects on CBF. When these effects are considered with its roles in maintaining the integrity of BBB and myelin (described in detail in the subsequent section), the magnitude of the potential impact of cerebral ECs on the pathological aetiology of CSVD can be fully appreciated. As for the causes of endothelial dysfunction, aging is the greatest risk factor for CSVD and a major contributor to endothelial dysfunction through several pathways involving adaptor protein p66*^Shc^*, oxidative stress described more thoroughly elsewhere (De Silva and Faraci, 2020). Additionally, the linkage between endothelial dysfunction and another important risk factor for CSVD, hypertension, is also established (Dharmashankar and Widlansky, 2010), involving excessive vascular oxidative stress, vascular inflammation and reduced prostaglandins productions (Dharmashankar and Widlansky, 2010; Cipolla et al., 2018).

### Blood-brain barrier breakdown

The BBB is primarily composed of ECs, the TJs between ECs, the basement membrane, astrocytic end-foot processes, and pericytes. It tightly regulates the movement of ions, molecules and cells to and from the circulating blood and the CSF or brain parenchyma, ensuring proper neuronal function as well as protecting the brain from harmful substances and pathogens. A breakdown of the BBB permits the extravasation of fluids and other plasma constituents, causing an enlargement of the perivascular space, localized damage to brain parenchyma such as cerebral microbleeds, and white matter changes (Hartz et al., 2012; Wardlaw et al., 2013b; Ihara and Yamamoto, 2016). Furthermore, the impairment might also increase the interstitial fluid volume and thicken and/or stiffen arteriole walls, exacerbating their vasodilatation and further compromising transport of oxygen and nutrient (Wardlaw et al., 2019). Evidence that impairment of BBB is a key contributing component of CSVD pathogenesis is accumulating (Wardlaw, 2010). In autopsy studies oedemas were observed in white matter lesions, indicating fluid leakage due to impaired BBB (Feigin and Popoff, 1963; Black et al., 2009). This result was corroborated by a study that used albumin extravasation to evaluate BBB integrity, wherein widespread leakage was found in the aging brain and accumulated in severe white-matter lesions (Simpson et al., 2007). Recently, dynamic contrast-enhanced (DCE)-MRI has emerged as a novel tool to quantitatively evaluate BBB permeability in patients. Using the DCE-MRI method, multiple studies revealed that BBB integrity was disrupted in CSVD patients and that the extent of increased BBB permeability was associated with a higher white matter hyperintensity burden as well as cognitive decline (Munoz Maniega et al., 2017; Zhang et al., 2017; Li Y. et al., 2018; Walsh et al., 2021). Similarly, a longitudinal study investigated by Wardlaw et al. (2013a) revealed a significant correlation between poor functional outcome and increased BBB permeability in CSVD patients.

With regard to possible causes of BBB disruption in CSVD, several potential mechanisms have been proposed (Figure 2). The formation and maintenance of TJ proteins claudin-5 and occludin, which are expressed by ECs, are vital to the integrity of the BBB. An *in vitro* study showed ECs dysfunction resulted in a reduction of claudin-5 expression and in animal models and patients with CSVD, and EC dysfunction corresponded to BBB breaches (Nitta et al., 2003; Bailey et al., 2011; Rajani et al., 2018). ECs dysfunction may also promote the degradation of the TJ proteins by releasing MMP such as MMP-2 and MMP-9 (Yong et al., 2001; Gheissari et al., 2018; Loso et al., 2018). Using rat models of CSVD, one study found an increase in expression of MMP-2 and MMP-9, and consequently, a decrease in claudin-5 and occludin, which was mitigated with treatment of MMP inhibitors (Yang et al., 2007). In addition, oxidative stress (i.e., increased reactive oxygen species) due to aging and hypertension in CSVD are considered major risk factors of BBB failure as it reduces NO bioavailability, resulting in a loss of TJ proteins (De Silva and Miller, 2016). Recent studies have presented other potential mechanisms that give rise to BBB dysfunction with aging. Acid sphingomyelinase (ASM) is a sphingolipid metabolizing enzyme, primarily derived from ECs in brain. Park et al. (2018) found increased ASM is a critical factor for BBB disruption. Genetic inhibition and endothelial-specific knockdown of ASM in mice ameliorated BBB breakdown. In contrast, overexpress of ASM in brain ECs accelerated BBB impairment and neurodegenerative change, suggesting a novel role for ASM in neurovascular function in aging (Park et al., 2018). The study also revealed that the increased permeability of the BBB associated with ASM expression is due to increased caveolae-mediated transcytosis, without detectable change in TJ proteins (Park et al., 2018). Transcriptional regulator sirtuin 1 is associated with endothelial dysfunction in aging. The declined expression of sirtuin 1 is linked to changes in permeability of the BBB in a study reported by Stamatovic et al. (Stamatovic et al., 2019). Conversely, the increased expression of sirtuin 1 protects against BBB impairment in aged transgenic mice (Stamatovic et al., 2019). The mechanism underlying sirtuin 1-meaiated BBB integrity is associated with stabilization claudin-5/ZO-1 interactions and the level of claudin-5 expression (Stamatovic et al., 2019). More recently, in a rat model of chronic cerebral hypoperfusion representing CSVD, Sun et al. found pericyte loss in the brain associated with the BBB impairment (Sun et al., 2021).

### White matter damage

WMH is a hallmark feature of human CSVD, predominantly observed around the ventricles and subcortically. The prevalence of WMH in MR images increases from about 5% for people aged 50 years to nearly 100% for people aged 90 years (Wardlaw, 2001). WMH is a well-established marker for predicting gait dysfunction *via* disruption of network and cortical thinning (de Laat et al., 2011; Kim et al., 2016). A wide range of evidence revealed WMH is associated with worse cognitive performance in CSVD (Debette and Markus, 2010; van der Holst et al., 2018; Jiang J. et al., 2022) and is due to disrupted structural and functional connectivity, as well as changes in cortical thickness at multiple cognitive domains (Righart et al., 2013; Tuladhar et al., 2015; Ter Telgte et al., 2018). Pathological changes of WMH are associated with several aspects of white matter damage including demyelination, loss of oligodendrocytes and axonal damage (Gouw et al., 2011). Both hypoperfusion and BBB impairment have been suggested as causes of WMH.

Compelling studies have observed hypoperfusion in the WMH using PET and MRI, likely resulting from vascular pathology involving multiple small arterioles (Moody et al., 1997; Markus et al., 2000; Thal et al., 2003; Makedonov et al., 2013; Wong et al., 2019). Interestingly, a comparative study found low CBF in normal-appearing white matter as well, to a lesser extent than that in the regions of WMH (O’Sullivan et al., 2002). Consistent with these findings, Promjunyakul et al. (2018) recently reported normal-appearing white matter with low CBF becoming abnormal at follow-up. The investigation also used diffusion tensor imaging (DTI) to evaluate the structural integrity of white matter. They concluded that CBF and DTI metrics were able to independently predict WMH growth and that WMH progression is likely due to demyelinating injury, secondary to insufficient perfusion (Promjunyakul et al., 2018). Although the etiology of impaired CBF in white matter still remains incomplete and unclear, ECs dysfunction-mediated pathological vasodilation is considered a pivotal contributor as we discussed earlier. More recently, a study using stroke-prone spontaneously hypertensive rat (SHR-SP) as a CSVD model showed that dysfunctional ECs secrete HSP 90α, which impeded oligodendroglial differentiation, thereby impairing myelination and myelin repair (Rajani et al., 2018). The authors also revealed that restoring EC function was able to reverse the oligodendroglia pathologies. Thus, this evidence supports EC dysfunction as one of the determinant contributors to white matter damage *via* vascular and non-vascular effects.

A compromised BBB is another pathology linked to the development of WMH (Figure 2). In an MRI study, BBB breakdown in the WMH was worse than that in the normal-appearing white matter and gray matter, evidenced by higher leakage rates and volumes (Wong et al., 2019). BBB impairment was aggravated in proximity to WMH. This finding supports previous studies in which an increase in BBB permeability near the WMH was found, implying that the BBB in normal appearing white matter is increasingly impaired in proximity to WMH (Huisa et al., 2015; Wardlaw et al., 2016; Munoz Maniega et al., 2017). Furthermore, it has been reported that MRI diffusivity is able to discriminate WMH from normal-appearing white matter, including mild WMH (Ihara and Yamamoto, 2016). Thus, BBB impairment might be an early predictor of white matter damage, occurring prior to the formation of WMH. Several hypotheses have been raised concerning the white matter damage resulting from leaky BBB. The impaired BBB may cause an increase in interstitial fluid and the resulting perivascular edema can intoxicate brain cells (Li Q. et al., 2018). Additionally, BBB breakdown allows for the entry of potentially harmful toxins and immune cells into the brain, which may directly cause deleterious effects (Sweeney et al., 2018). The leaky constituents can indirectly cause brain injury as well. For instance, the extravasated fibrinogen obstructs the maturation of oligodendrocyte precursor cells, thereby inhibiting myelin maintenance and repair (Wardlaw et al., 2019). Fibrin cleaved from fibrinogen is able to trigger local neuroinflammation by activating microglia and recruiting peripheral macrophages, cells known to contribute to the progression of demyelination (Petersen et al., 2018; Sweeney et al., 2018). Although evidence supports BBB breakdown playing a pivotal role in WMH formation, it is important to note that the BBB can remain intact in patients with WMHs, as exemplified by Rajani et al. (2019). Likewise, Hainsworth et al. (2017) corroborated a lack of direct association of BBB dysfunction with white matter abnormalities. Therefore, further research in this area is required to elucidate this important relationship.

### Inflammation

There is growing interest in exploring the relationship between inflammation and CSVD. Based on circulating biomarkers, CSVD inflammation is classified as either systemic inflammation (e.g., C-reactive protein, interleukin-6) or vascular inflammation (e.g., homocysteine, vWF) (Poggesi et al., 2016; Low et al., 2019). Interestingly, regional analysis showed vascular and systemic inflammation appears to correspond to two subtypes of CSVD with different site preferences (Low et al., 2019). Vascular inflammation is associated with the formation of CSVD in brain regions supplied by deep perforator arteriopathy (DPA) (e.g., basal ganglia) (Low et al., 2019; Li T. et al., 2020), while systemic inflammation is often linked to cerebral amyloid angiopathy (CAA)-related vascular injury in brain regions supplied by cortical and leptomeningeal vessels (Low et al., 2019; Li T. et al., 2020). One possible explanation for the differential vulnerability to vascular damage is due to distinct features of the cerebrovascular network in certain regions (Low et al., 2019). Although the link between CSVD subtype and inflammation classification is unclear, evidence supports inflammation’s involvement in critical pathophysiological mechanisms of endothelial dysfunction and BBB disruption. In the spontaneously hypertensive rat (SHR) CSVD animal model, Kaiser et al. (2014) showed peripheral immune cell (e.g., T cells, NK cells) migration and microglial activation (e.g., IL-1β secretion), in conjunction with inflammation, endothelial dysfunction and BBB disruption. In the same CSVD model, Zhang et al. (2019). observed cognitive function of the SHR animals concomitantly with a high expression of Toll-like receptor 4 (TLR4) in the hippocampus, which is a key signal transduce to trigger inflammatory responses. Consistently, Gao et al. (2019) not only confirmed the high expression of TLR4 in hippocampus but also glial (microglia and astrocyte) activation and evaluated level of IL-1β and TNF-α. The study also found white matter degeneration in corpus callosum and external capsule of SHR. In the SHR-SP model, Jalal et al. (2015) reported infiltrating T cells and neutrophils appeared around endothelial cells and BBB leakage was observed subsequently. The animals exhibited extensive white matter abnormalities and behavioral decline, which were effectively restored by treatment with anti-inflammatory agent minocycline. Brain-gut axis refers to the bidirectional signaling between the gastrointestinal tract, or gut microbiota, and the brain (Dinan and Cryan, 2017). The axis has gained growing interest in recent years for its role in maintaining homeostasis. Accumulating evidence suggest a link between the gut microbiome and the development of cardiovascular diseases and neurodegenerative disorders (Karlsson et al., 2012; Friedland, 2015; Durgan et al., 2019; Sanchez-Rodriguez et al., 2020). Using SHR-SP model, James et al. (Nelson et al., 2021). provided direct evidence that the gut microbiome involved in the onset or progression of CSVD *via* influencing the integrity of BBB. The possible mechanism is that microbiome enhanced inflammation in the gut, which may become systemic, eventually inducing neuroinflammation *via* the brain-gut axis. A medical research council cognitive function and aging study also revealed the elevation of inflammatory markers in close proximity to diseased arteries accompanied by BBB dysfunction (Wharton et al., 2015). A more recent clinical study found an association between neutrophil count and CSVD, especially in enlarged perivascular spaces within the basal ganglia and in lacunes, supporting its value for predicting the presence of CSVD (Jiang L. et al., 2022). Possible mechanisms of inflammation in CSVD development include in endothelial dysfunction, white matter lesions and BBB disruption (Jiang L. et al., 2022). The relationship between inflammation and BBB impairment is well-documented; however, the direction of causality remains debatable. Several studies suggest that BBB damage precedes inflammation, enabling entry of detrimental plasma components and immune cells (Petersen et al., 2018; Sweeney et al., 2018). Others believe that inflammation compromises BBB integrity, which can be preserved by pharmacologically blocking the inflammation (Abbott, 2000; Yenari et al., 2006; Rouhl et al., 2012; Kerkhofs et al., 2020). More recently, Walsh et al. (2021), using PET imaging of the translocator protein ^11^C-PK11195, found both microglial activation and increased BBB permeability occurred in sporadic CSVD, but they were spatially distinct processes. A possible explanation for this finding is that these two processes might occur independently of each other (Edison, 2021). Thus, to reveal the sequence of BBB and inflammation in CSVD, longitudinal studies with multiple time points would be valuable.

**FIGURE 3:**
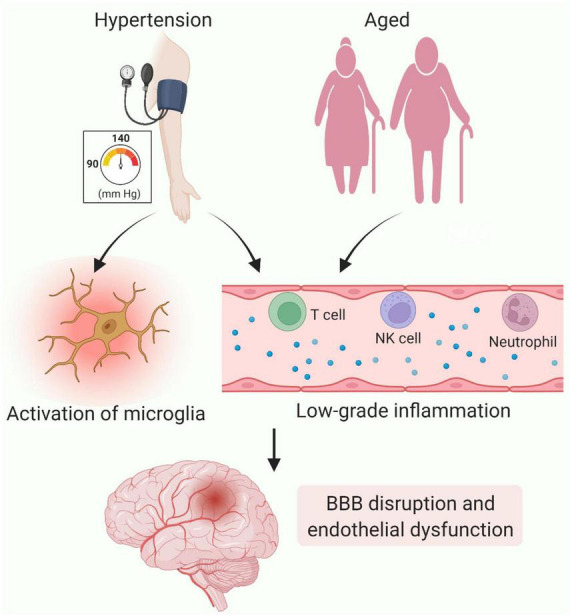
Contributions of inflammation to cerebral small vessel disease (CSVD). Chronic hypertension-induced vascular remolding leads to hypoxia and ischemia of local brain parenchyma, thereby triggering activation of microglia. In addition, hypertension and aging can generate chronic, low-grade inflammation. The inflammatory molecules generated and immune cells recruited as a result contribute to BBB impairment and endothelial dysfunction. Figure created using BioRender.com.

Other potential risk factors of CSVD inflammation have gained attention as well (Figure 3). Hypertension and advanced age are well-known epidemiologic risk factors for CSVD (Schreiber et al., 2020). They are the major contributors to the pathologic changes of cerebral small vessels, characterized by loss of smooth muscle cells from the tunica media, deposits of fibro-hyaline material, narrowing of the lumen, and thickening of the vessel wall (Pantoni, 2010). Ultimately, the vascular remodeling/injury led to impaired CBF regulation and subsequent hypoxia and ischemia of local brain parenchyma (Evans et al., 2021). These histopathological changes are recognized features of CSVD development. In addition to inducing vascular alterations, hypertension and aging can also cause endothelial dysfunction, BBB breakdown in CSVD as described in previous sections. Recently, hypertension has been proposed to trigger low grade systemic and vascular inflammation as well as activation of microglia, both which are also associated with CSVD (Petrie et al., 2018; Evans et al., 2021). Age-related inflammation, termed inflammaging, is an emerging concept that refers to the status of chronic, sterile, low-grade inflammation in older organisms, resulting from cellular senescence, immunosenescence, mitochondrial dysfunction, defective autophagy, and metaflammation (Franceschi et al., 2000; Vitale et al., 2013). Inflammaging is increasingly considered a risk factor for CSVD, especially for age-related CSVD. A recent review from Li T. et al. (2020) proposed inflammaging might contribute to CSVD progression by inducing systemic and vascular inflammation. They suggested multiple inflammatory mediators such as tumor necrosis factor (TNF), caspase-1, IL-1β, and NOD-like receptor family pyrin domain containing 3 (NLRP3) inflammasome are produced during inflammaging, thereby resulting in BBB leakage and endothelial dysfunction.

### Degeneration of smooth muscle cells

In CSVD, progressive degeneration and loss of smooth muscle cells are characteristic alterations that contribute to the vascular remodeling and the subsequently impaired regulation of CBF (Frosen and Joutel, 2018; Evans et al., 2021). Remodeling is often coupled with infiltration of plasma components or lipids in the vessel wall, which may further exacerbate vascular alteration and dysfunction. It has been reported that macrophages infiltrating vascular walls produce reactive oxygen species, provoke the hypertrophy of smooth muscle cells, and secrete MMPs (Virdis et al., 2004). Additionally, the infiltrating fibrinogen is able to inhibit the expression of peroxisome proliferators-activated receptors in smooth muscle cells, resulting in an increased expression of C-reactive protein and MMP-9 and an accelerated progression of atherosclerotic plaques (Wang et al., 2015). In mature arteries, smooth muscle cells maintain contractile phenotype with a low synthetic activity. In response to local environmental changes from injury or hypertension, the cells can convert to synthetic phenotype with decreased contractility and increased proliferation. This phenotypic conversion of smooth muscle cells was recently considered another pathological character of vascular remodeling in CSVD. Liu et al. (2021) using the SHR CSVD model revealed that smooth muscle cells in cerebral small arteries shifted from the contractile phenotype to synthetic phenotype during the chronic process of hypertension and aging. Li C. C. et al. (2020) successfully established a mouse CSVD model using angiotensin II, wherein BBB dysfunction, white matter damage and cerebral vascular remodeling (e.g., thickening of vascular wall) were found. The study also observed low expression of the contractile type marker α-smooth muscle actin (α-SMA) and high expression of high expression of the synthetic phenotype marker proliferating cell nuclear antigen (PCNA), indicating the phenotypic conversion of smooth muscle cells. The exact mechanism of smooth muscle cells degeneration is not yet well understood. It has been showed that amyloid ß (Aß) peptide deposition might be responsible in CAA (Frosen and Joutel, 2018). Interestingly, in SHR-SP, impaired Aß clearance was observed as well (Held et al., 2017), implicating a role in smooth muscle cells degeneration in non-amyloid CSVD. Nevertheless, future studies are required to elucidate the mechanisms of smooth muscle cells degeneration at in CSVD.

### Prevention and treatment for cerebral small vessel disease

Prevention and treatment of CSVD include lifestyle modifications such as smoking and pharmacologic interventions. Antiplatelet, anti-hypertensive and statin therapies are standard pharmacologic treatments (Cannistraro et al., 2019). Anti-platelets are generally used for ischemia stroke. A pooled analysis of randomized trials has shown that antiplatelet therapy such as aspirin after acute subcortical infarction reduces the risk of recurrent stroke by 30% (Kwok et al., 2015). A meta-analysis of four trials (ACCORD-MIND, PRoFESS, PROGRESS, and SCOPE) showed that intensive antihypertensive medication resulted in significantly less progression of WMH compared to guidelines for blood pressure reduction (van Middelaar et al., 2018). Statins are another evidence-based therapy for CSVD. Less WMH progression in patients who took low dose rosuvastatin than in control, suggesting statin might delay WMH progression (Ji et al., 2018). Another randomized controlled trial found low-dose statins before stroke reduced post-stroke WMH progression (Xiong et al., 2014). However, data from Prevention of Decline in Cognition after Stroke Trial (PODCAST) showed that neither intensive lipid-lowering nor blood pressure ameliorated the cognitive decline after stroke delay (Bath et al., 2017). Apparently, standard pharmacologic treatments are beneficial for CSVD treatment, but their efficacy is limited. Encouragingly, with improved understanding of the pathogenesis of CSVD, novel therapeutic interventions have been proposed. In an animal study, cilostazol, a phosphodiesterase inhibitor, reduced cognitive decline and ameliorated gliovascular damage *via* endothelial stabilization (Kitamura et al., 2017). The lacunar intervention (LACI-1 and LACI-2) Trials testing cilostazol for CSVD are ongoing (Blair et al., 2019; Wardlaw et al., 2020). Minocycline is an anti-inflammatory drug with multiple immune-modulating properties with promising clinical data for multiple sclerosis (Brundula et al., 2002; Metz et al., 2017). In CSVD models, Jalal et al. (2012, 2015) showed minocycline decreased neuroinflammation, alleviated white matter damage, improved behavioral performance, and prolonged life expectancy. Several other anti-inflammatory drugs including fingolimod, natalizumab and rituximab have been utilized to treat neuroinflammatory diseases and they are expected to be potent candidates for CSVD treatment (Fu and Yan, 2018).

Cell therapy is widely viewed as a promising strategy for treatment of neurological disorders. Currently, there is a considerable interest in stem-cell therapy for CSVD treatment. Nakazaki et al. (2019) reported the first study investigating the therapeutic potential of mesenchymal stem cells (MSCs) for improving cognitive impairment in a CSVD model of SHR. The study revealed that intravenously infused MSCs restored BBB function by remodeling the microvasculature and inhibited progressive brain atrophy by reducing Aβ accumulation. As a result, improved cognitive function was achieved in the animals. Transplantation of MSCs was also found to increase the density of the pial microvascular network in the SHR brain, reaching a similar level as young Wistar-Kyoto rats (Sokolova et al., 2017). This finding implies that MSCs may have the potential to improve hypoperfusion resulting from the low CBF in CSVD. Given the above-mentioned contributions of white matter damage to CSVD development, white matter might be a compelling therapeutic target. In fact, several stem cell populations capable of differentiating into oligodendrocytes, e.g., oligodendrocyte progenitor cells and glial-restricted progenitors (GRPs), have been found to myelinate in the adult brain, resulting in both structural repair and functional restoration of damaged white matter (Piao et al., 2015; Li S. et al., 2019; Wang et al., 2021). Thus, transplantation of myelinated stem cells for CSVD treatment is worth evaluation in future research.

## Conclusion

Cerebral small vessel disease is an important pathology of stroke, age-related cognitive decline, accounting for about half of all dementias. Controlling traditional risk factors *via* pharmacologic treatments and lifestyle modification are the current approaches for prevention and therapy of CSVD, particularly for patients with clinical presentations. Covert cerebral small vessel disease (ccSVD) is detectable on neuroimages lacking overt neurological manifestations, is highly prevalent in aging population and increases the risk of future stroke, dementia or death (Wardlaw et al., 2021; Bordes et al., 2022). A recent guideline released from European Stroke Organization (ESO) suggested that antiplatelet drugs such as aspirin are not recommended and little evidence is found for lipid lowering in ccSVD (Wardlaw et al., 2021). Thus, there is an urgent need to explore novel avenues to stop the development and clinical diseases resulting from ccSVD. The incomplete understanding of pathogenesis is a major reason for the lack of more specific preventive and therapeutic strategies for CSVD. Encouragingly, accumulating evidence supports pathophysiological changes such as endothelial dysfunction, white matter abnormality, and BBB impairment, as well as inflammation, are responsible for CSVD etiology. Such advancements in the understanding of CSVD has provided targets for potential CSVD remedies. Studies targeting endothelia and inflammation have obtained positive results in experimental studies. For exploring more potential targets and hastening their clinical translation, many questions remain to be addressed in future studies, for instance the order of and interactions between these pathological changes. Additionally, the initiator of CSVD presence is still unclear, whose identification might allow the possibility for a reversal in the earliest stages.

## Author contributions

YG, DL, DC, JL, JM, SL, and CC conceived and designed the manuscript. YG, AT, and CC wrote the main manuscript and revised the manuscript. All authors reviewed the manuscript, read and approved the final manuscript.
